# CD4/CD8 imbalance as a key factor in the progression from mogamulizumab-induced drug eruption to adult T-cell leukemia/lymphoma

**DOI:** 10.1016/j.jdcr.2025.05.011

**Published:** 2025-06-10

**Authors:** Sakiho Inayoshi, Takuya Inoue, Kazunari Sugita

**Affiliations:** Division of Dermatology, Department of Internal Medicine, Faculty of Medicine, Saga University, Saga, Japan

**Keywords:** adult T-cell leukemia/lymphoma, double immunofluorescence staining, drug eruption, human T-cell leukemia virus type 1, mogamulizumab

## Introduction

Adult T-cell leukemia/lymphoma (ATLL) is a malignancy of CD4^+^ T cells caused by human T-cell leukemia virus type 1, characterized by varying subtypes and a poor prognosis.^1^^,^^2^ As most ATLL tumor cells express CC chemokine receptor 4 (CCR4), mogamulizumab, a monoclonal antibody targeting CCR4, was developed as a therapy. While effective, mogamulizumab is frequently associated with skin-related adverse events, including erythema multiforme, Stevens-Johnson syndrome, and toxic epidermal necrolysis.^3^^,^^4^ These eruptions may persist, complicating clinical management. Moreover, distinguishing between ATLL-related skin infiltrates and mogamulizumab-induced eruptions poses a diagnostic challenge. Here, we present a case where mogamulizumab initially induced a drug-related rash, later followed by ATLL-specific skin lesions.

## Case report

**Fig 1:**
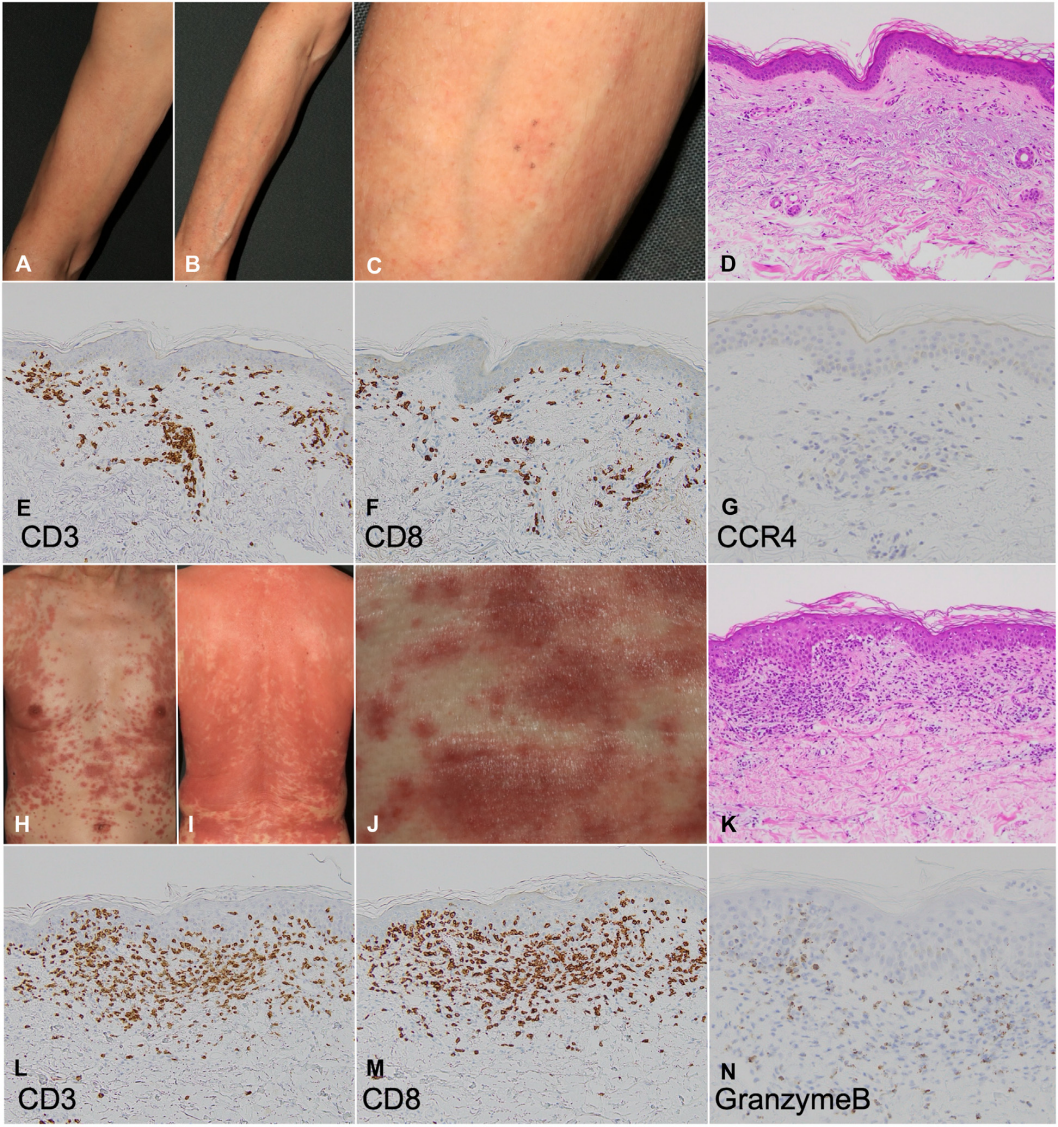
**A** and **B,** Clinical images of the upper extremities. **C,** The lesion on the left forearm from which a skin biopsy was taken. **D,** Hematoxylin and eosin (H&E) staining, ×200. **E,** CD3 staining, ×200. **F,** CD8 staining, ×200. **G,** CCR4 staining, ×400. **H** and **I,** Clinical images of the trunk. **J,** The lesion on the left flank from which a skin biopsy was taken. **K,** Hematoxylin and eosin (H&E) staining, ×200. **L,** CD3 staining, ×200. **M,** CD8 staining, ×200. **N,** Granzyme B staining, ×400.

A 71-year-old male with polymyalgia rheumatica and paroxysmal atrial fibrillation was diagnosed with ATLL and initiated on mogamulizumab and CHOP chemotherapy (cyclophosphamide, doxorubicin, vincristine, and prednisolone). Mogamulizumab was administered at a dose of 50 mg (1 mg/kg) on day 1. Blood tests revealed an interleukin (IL)-2 receptor level of 6155 U/ml (reference range: 122-496 U/ml) and a lactate dehydrogenase (LDH) level of 249 U/l (reference range: 124-222 U/l). Three weeks after treatment initiation, infiltrative erythema developed on the upper extremities, lower abdomen, and lumbar region (Fig 1, *A* and *B*). Given the clinical timeline, drug eruption was suspected. A biopsy from an erythematous lesion on the left forearm (Fig 1, *C*) revealed interface dermatitis with liquefaction degeneration and perivascular lymphocyte and eosinophil infiltration in the superficial dermis (Fig 1, *D*). Immunohistochemistry showed that infiltrating immune cells were primarily CD3^+^ T lymphocytes, mainly CD8^+^, with few CCR4-positive cells, suggesting drug eruption (Fig 1, *E-G*). The patient was also taking bisoprolol, trimethoprim-sulfamethoxazole, and famotidine. These medications were discontinued, and prednisolone 25 mg/day was started. Notably, mogamulizumab had been administered 4 weeks prior to the discontinuation of these drugs and was not re-administered thereafter. However, the erythematous lesions continued to spread, developing into infiltrative plaques on the trunk (Fig 1, *H* and *I*). A subsequent biopsy from the left flank (Fig 1, *J*) showed dense lymphocytic infiltration in the superficial dermis, with some cells extending into the epidermis (Fig 1, *K*). Immunohistochemistry confirmed CD3^+^-predominant infiltration with CD8^+^ interface dermatitis, leading to a diagnosis of drug eruption attributed to mogamulizumab (Fig 1, *L* and *M*). A few granzyme B-positive cells were also present (Fig 1, *N*). The prednisolone dose was subsequently increased to 60 mg/day. Following treatment and steroid escalation, the IL-2 receptor level declined to 1904 U/ml and LDH to 194 U/l, suggesting partial improvement. The erythema initially improved but exacerbated twice while tapering prednisolone to 40 mg/day.

**Fig 2:**
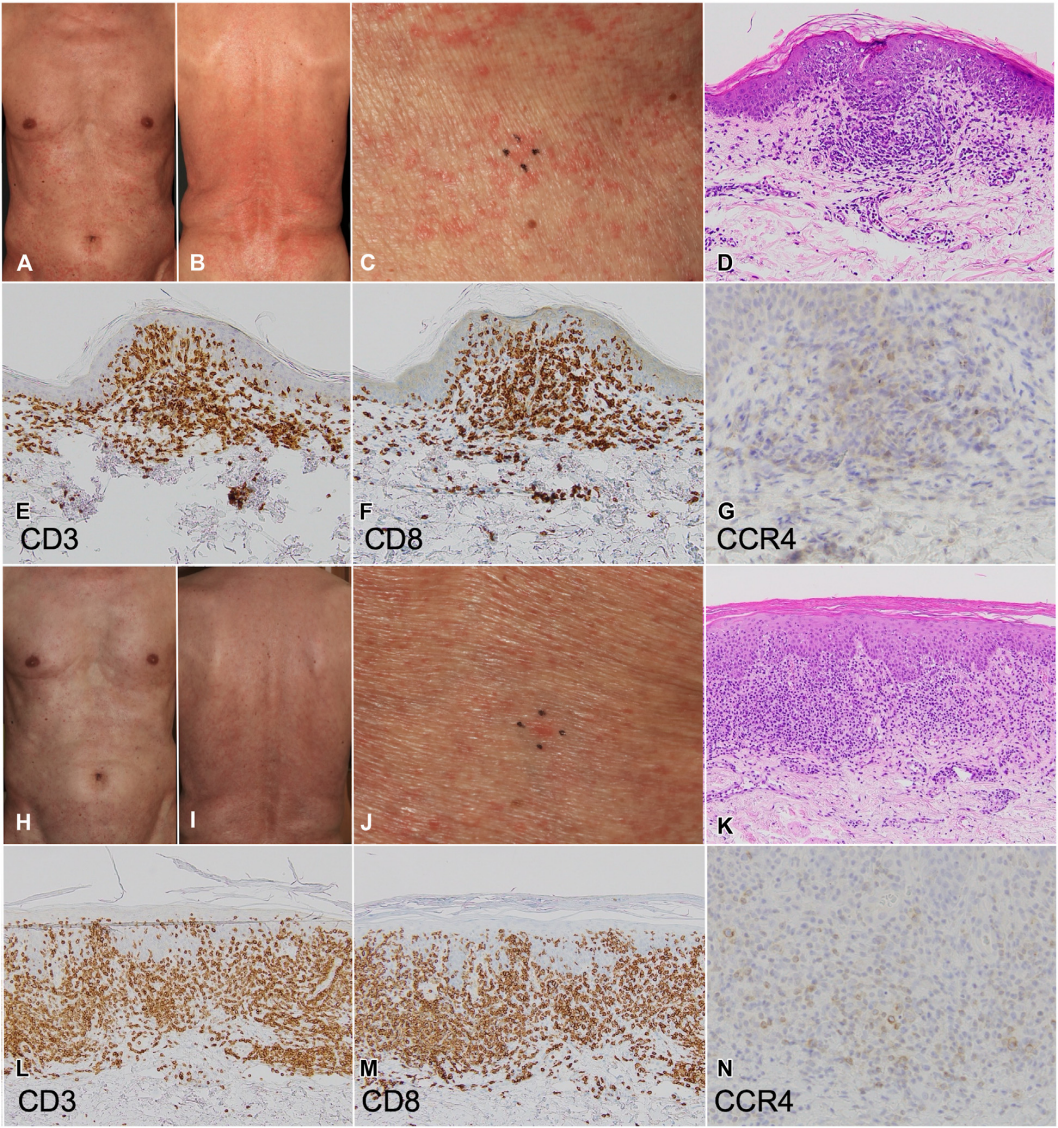
**A** and **B,** Clinical images of the trunk. **C,** The lesion on the left abdomen from which a skin biopsy was taken. **D,** Hematoxylin and eosin (H&E) staining, ×200. **E,** CD3 staining, ×200. **F,** CD8 staining, ×200. **G,** CCR4 staining, ×400. **H** and **I,** Clinical images of the trunk. **J,** The lesion on the lower back from which a skin biopsy was taken. **K,** Hematoxylin and eosin (H&E) staining, ×200. **L,** CD3 staining, ×200. **M,** CD8 staining, ×200. **N,** CCR4 staining, ×400.

One month later, the first exacerbation occurred, with infiltrative erythema developing on the lower abdomen and back (Fig 2, *A* and *B*), and the IL-2 receptor level rising to 3578 U/ml and LDH to 227 U/l. A biopsy from the left abdomen showed medium- to large-sized lymphocytes infiltrating the superficial dermis, with some demonstrating epidermotropism (Fig 2, *C* and *D*). Immunohistochemistry showed that most immune cells were CD3^+^ and CD8^+^, with a few CCR4-positive cells (Fig 2, *E-G*). These findings did not exclude cutaneous ATLL infiltration. Prednisolone was increased to 60 mg/day but gradually tapered to 40 mg/day over 3 weeks, leading to a second exacerbation.

Erythematous papules and nodules expanded on the trunk (Fig 2, *H* and *I*), and the IL-2 receptor level and LDH further increased to 11370 U/ml and 317 U/l, respectively, indicating ongoing or progressive disease activity. A biopsy from the lower back (Fig 2, *J*) showed lymphocytic infiltration with small- to medium-sized atypical nuclei. Immunohistochemistry confirmed CD3^+^ and CCR4^+^ atypical lymphocytes, with CD8^+^ lymphocytes suggesting an immune response against tumor cells (Fig 2, *L-N*). Elevated serum IL-2 receptor levels and human T-cell leukemia virus type 1 proviral DNA detection in the skin confirmed cutaneous ATLL infiltration. CHOP therapy was resumed, leading to gradual resolution of erythema, suggesting CHOP itself is not associated with drug eruptions.

**Fig 3:**
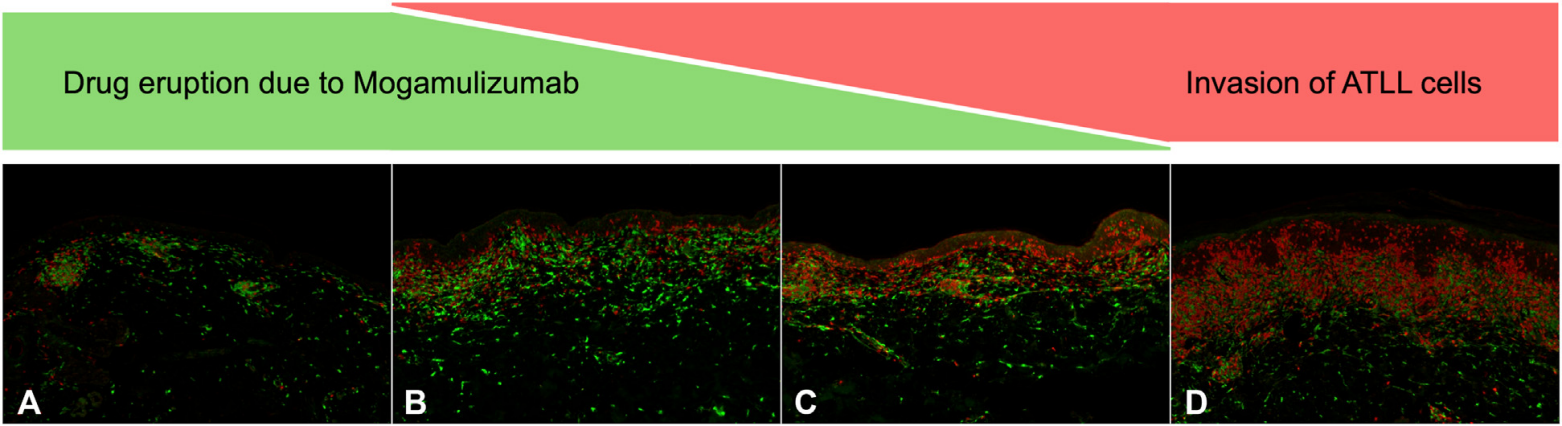
Double immunofluorescence staining for CD4 and CD8 in a chronological series of 4 skin biopsies, ×200. CD8^+^ cells are labeled with green fluorescence (fluorescein isothiocyanate), and CD4^+^ cells are labeled with red fluorescence (phycoerythrin). **A,** First biopsy, corresponding to Fig 1, *D*. **B,** Second biopsy, corresponding to Fig 1, *K*. **C,** Third biopsy, corresponding to Fig 2, *D*. **D,** Fourth biopsy, corresponding to Fig 2, *K*. *ATLL*, Adult T-cell leukemia/lymphoma.

To assess CD4^+^ and CD8^+^ distribution, double immunofluorescence staining was performed on 4 biopsies from the lesions shown in Figs 1, *C* and *J*, 2, *C* and *J*. Staining demonstrated a shift from CD8^+^ T-cell predominance in early biopsies to CD4^+^ predominance over time (Fig 3). These findings suggest that the initial lesions were caused by a mogamulizumab-induced drug eruption, which subsequently progressed to ATLL infiltration.

## Discussion

Drug eruptions and cutaneous ATLL share similar clinical features, making their distinction challenging.^5^ In this patient, the rash worsened despite discontinuation of trimethoprim-sulfamethoxazole, famotidine, and bisoprolol, ruling out these medications. Mogamulizumab often causes prolonged drug eruptions that persist postdiscontinuation, complicating diagnosis.^6^ Furthermore, as observed in this case, skin lesions during mogamulizumab therapy can signify ATLL-specific cutaneous infiltration, further complicating differentiation.

Mogamulizumab, a CCR4^+^-targeting monoclonal antibody, is effective in treating ATLL but frequently causes immune-related adverse events, particularly drug eruptions. Mogamulizumab-induced drug eruptions typically present as persistent rashes, requiring prolonged management. In this case, the initial rash exhibited CD8^+^ T-cell predominance, supporting a diagnosis of drug eruption. However, the subsequent emergence of CD4^+^ predominance and lesion progression raised suspicion of ATLL infiltration. This highlights the overlapping features of mogamulizumab-induced drug eruptions and ATLL-related lesions, emphasizing the need for repeated clinical and pathological evaluations.

Although ATLL is typically characterized by CD4^+^ T-cell infiltration, rare cases of CD8^+^ T-cell predominance have been reported.^7^ Considering this, we performed CD4/CD8 double staining to assess epidermotropism and cell distribution. As demonstrated in Fig 3, modulation of the CD4/CD8 ratio emerged as a critical marker for distinguishing between drug eruption and ATLL-specific skin infiltration. Repeated biopsies and immunohistochemistry revealed lesion evolution. Specifically, the shift from CD8^+^ to CD4^+^ predominance over time, along with CD4^+^ epidermotropism, indicated ATLL progression. Additionally, CCR4 staining and human T-cell leukemia virus type 1 proviral DNA detection in later biopsies confirmed ATLL-specific cutaneous infiltration.

Taken together, this case is notable for illustrating the chronological evolution of immune cell profiles in the skin, transitioning from CD8^+^-dominant drug eruption to CD4^+^-predominant ATLL infiltration, as demonstrated by serial biopsies and CD4/CD8 double staining. Such longitudinal immunophenotypic analysis may aid in distinguishing reactive drug eruptions from early neoplastic infiltration in ATLL patients treated with mogamulizumab.

## Conflicts of interest

None disclosed.
